# Practical Experience in the Use of Oxy-Phosphate Cements

**Published:** 1885-02

**Authors:** W. D. Miller

**Affiliations:** Berlin


### Independent Practitioner.

Vol. VI. February, 1885. No. 2.

### Original Communications.

### PRACTICAL EXPERIENCE IN THE USE OF OXY-PHOSPHATE CEMENTS.

BY DR. W. D. MILLER, BERLIN.

On beginning the practice of dentistry in Berlin in the spring of 1879, I found a filing material extensively used by German dentists which I had never met with in America.

It at once attracted my attention on account of its superior hardness and apparent greater durability; it was also very highly commended by different practitioners with whom I spoke concerning it. This material was the now well-known oxy-phosphate cement.

It possessed a very evident advantage, in that it could be had in all possible desirable shades of color.

I immediately set about to determine by experiment the comparative solubility of the new material and the older oxy-chlorides in various solvents which were likely to be found it the human mouth. The results of these experiments were presented at the Lucerne meeting of the American Dental Society of Europe, in 1880. I bored holes, all of the same size, in strips of hard wood or ivory, filled them with the various cements to be tested, and immersed the strips in solutions of various organic acids, also in mixtures of saliva and bread, saliva and sugar, etc., etc.

The result was the same in all cases.

The oxy-phosphates, Poulson, Wolff, Lorenz, Rostaigne, etc., withstood the action of the solvent about equally well, and far better than any of the oxy-chlorides.

This is a result which I have also found to be justified by practical experience, as I can show fillings to-day which have been in the mouth over five years, and are still holding well.

One other result of these experiments was to induce me to use the oxy-phosphates to some extent in my practice, an unfortunate result, for I am now convinced that of all materials put into the hands of dentists for filling teeth, the oxy-phosphates are the most unreliable and treacherous. We sometimes see fillings so situated that they are constantly kept clean, which have stood the test for years with scarcely any alteration; while, perhaps, in the very next mouth that we examine, we will find fillings which were inserted only a few weeks previous, crumbling to pieces. Even after I had had a number of failures I was not willing to give up the material entirely. I held to the view that the fault was not in the material, but in the operator, and that greater care in mixing the cement, greater care in inserting it, excluding every trace of moisture, and greater care in properly finishing the filling, would insure uniform and good results.

I now know that the fault lies, in a great part at least, with the material. If the manufacturer succeeds in producing a good article, which is not by any means always the case, it being sometimes necessary to call back a "batch" after it has been distributed among the consumers, and if the operator succeeds in keeping it under such conditions that it does not attract too much moisture, and if he by chance mixes it in just the proper proportions to give the best results, and inserts it and finishes the filling with the proper amount of care, under complete exclusion of moisture, then, in crown cavities, it *may* in some mouths last four or five years, or even longer. The filling should always be examined a few days after insertion, to determine whether it has properly hardened, and in no case can it be guaranteed to last a certain number of years, or even months.

In all those cases where a filling of the nature of cement seems most to be indicated, in extensive cavities, on the approximal surfaces of bicuspids and molars, nearing the pulp, it has proved a failure, unless employed as a temporary filling, and even then its value is very questionable.

If we make, with all due caution, a filling of oxy-phosphate on the approximal surface of a bicuspid, tooth very soft, decayed under the gum, and pulp nearly exposed, the chances are, that at a time by no means very far off, the patient returns with an aching tooth; we find a large cavity under the margin of the gum, and an exposed, inflamed pulp. In such cases cement should not be used, even for temporary purposes, unless the neck of the tooth is protected by a layer of gutta-percha.

This result is brought about, not by the disintegration of the cement alone, but secondary caries seems to progress with considerable rapidity, because the antiseptic property of the filling material is almost zero, and because the concavity produced by the dissolution of the cement, as well as the roughened surface of the same, retains particles of food, and possibly because the oxy-phosphates contain substances well suited to the wants of the micro-organisms of decay.

I do not say that the result will *always* be as I have indicated. I have seen many notable exceptions, but it will be so in such a large proportion of cases as to render it entirely unsafe to place any confidence in a filling of oxy-phosphate; so that now, after an extensive experience in the use of this material, I have abandoned it altogether, except for upper front teeth, where the patient objects to the use of gold on account of its appearance; or, occasionally, for temporary teeth and for setting pivot teeth.

As a capping for exposed pulps, oxy-phosphates have, in my experience, proved an equal failure. Personally I have never made use of it for this purpose, but I have had abundant opportunity to watch its effect when employed by others, and the number of fillings that I have been obliged to remove on account of the supervention of pulpitis and pericementitis is sufficiently great to render its use in my own practice unjustifiable.

Especially where it is necessary, for some cause or other, to leave decayed or softened dentine in the cavity, oxy-phosphates should be avoided, as the smallest amount of moisture in the dentine prevents the thorough adaptation of the cement to the tooth structure; we do not secure a perfectly water-tight filling, and secondary decay is likely soon to follow. For all such cases (incisors and anterior surface of cuspids excepted) a filling combined of tin and gold is far better, and, indeed, it would be difficult to find a case where oxy-phosphates may be used which could not be treated to much greater advantage with the Abbot combination filling, and in a great many cases the latter may be used with success where oxy-phosphates are altogether out of the question.

The latter, undoubtedly, when properly hardened, resist the action of the fluids of the mouth much better than the other cements now in use, but their susceptibility to the action of moisture while in the soft state, their inequality and their lack of antiseptic power, very materially diminish their usefulness.

We need, at present, in a cement, a material which may be used in very soft, porous teeth, sometimes over partially decalcified moist dentine, and which will not only stop the decomposition of the softened dentine under the filling, but will also effectually shut out external agents, and at the same time, if possible, incite the underlying dentine to a recalcification. In all these points the oxy-phosphates are wanting, besides giving very inconstant results, even in more favorable cases.

There will, no doubt, be some who will not agree with me in placing so low an estimate upon these cements. Two years ago I should myself have expressed a very different opinion of them. Nor would I attempt to dissuade any one who chooses to test the matter in his own practice from so doing, since it is only by the combined experience of many that the whole truth may be reached.

